# Associations between older adults’ attitudes to ageing, mental health, and quality of life: a systematic review

**DOI:** 10.1007/s10433-026-00932-x

**Published:** 2026-07-09

**Authors:** Margot Juliëtte Overman, Lucy Hopkins, Kathryn Evans

**Affiliations:** Oxford Institute of Clinical Psychology Training and Research, University of Oxford, Warneford Hospital, Headington, Oxford, OX3 7JX UK

**Keywords:** Depression, Anxiety, Quality of life, Attitudes to ageing, Older adults, Systematic review

## Abstract

The stereotype embodiment theory posits that negative age stereotypes, which are not self-relevant in early life, are internalised at a young age. Cognitive models suggest that such internalised attitudes present a latent vulnerability which can be activated with increasing age and contribute to mental health difficulties. Attitudes to ageing are proposed to be multidimensional, with individual domains exerting differential effects on health outcomes. This review therefore aimed to assess the relationship of general and domain-specific attitudes to ageing with depression, anxiety, and quality of life (QoL) in older adults. The protocol was preregistered on PROSPERO (CRD42024537940). PsycInfo, Embase, Medline, CINAHL, Scopus, and Web of Science databases were searched from inception to February 7, 2026. 22 articles including 41,882 participants were identified and synthesised using a narrative approach, with quality assessed using the AXIS tool. Papers investigated depression (*n* = 11), QoL (*n* = 10), and anxiety (*n* = 7). General negative attitudes to ageing were associated with increased psychopathological symptoms and lower QoL. Across individual domains, negative attitudes to psychosocial loss were most reliably linked with poorer mental health. Conversely, positive attitudes to psychological growth may be a protective factor. These findings are consistent with the stereotype embodiment theory and suggest that attitudes to ageing may present a novel intervention target. Interpretations are limited by the observational nature of the studies, and a priority for future research is the use of experimental approaches to clarify the causality of observed associations and clinical application of attitudes to ageing as a treatment target for older adults’ mental health.

The online version contains supplementary material available at https://doi.org/10.1007/s10433-026-00932-x.

## Introduction

**Fig. 1:**
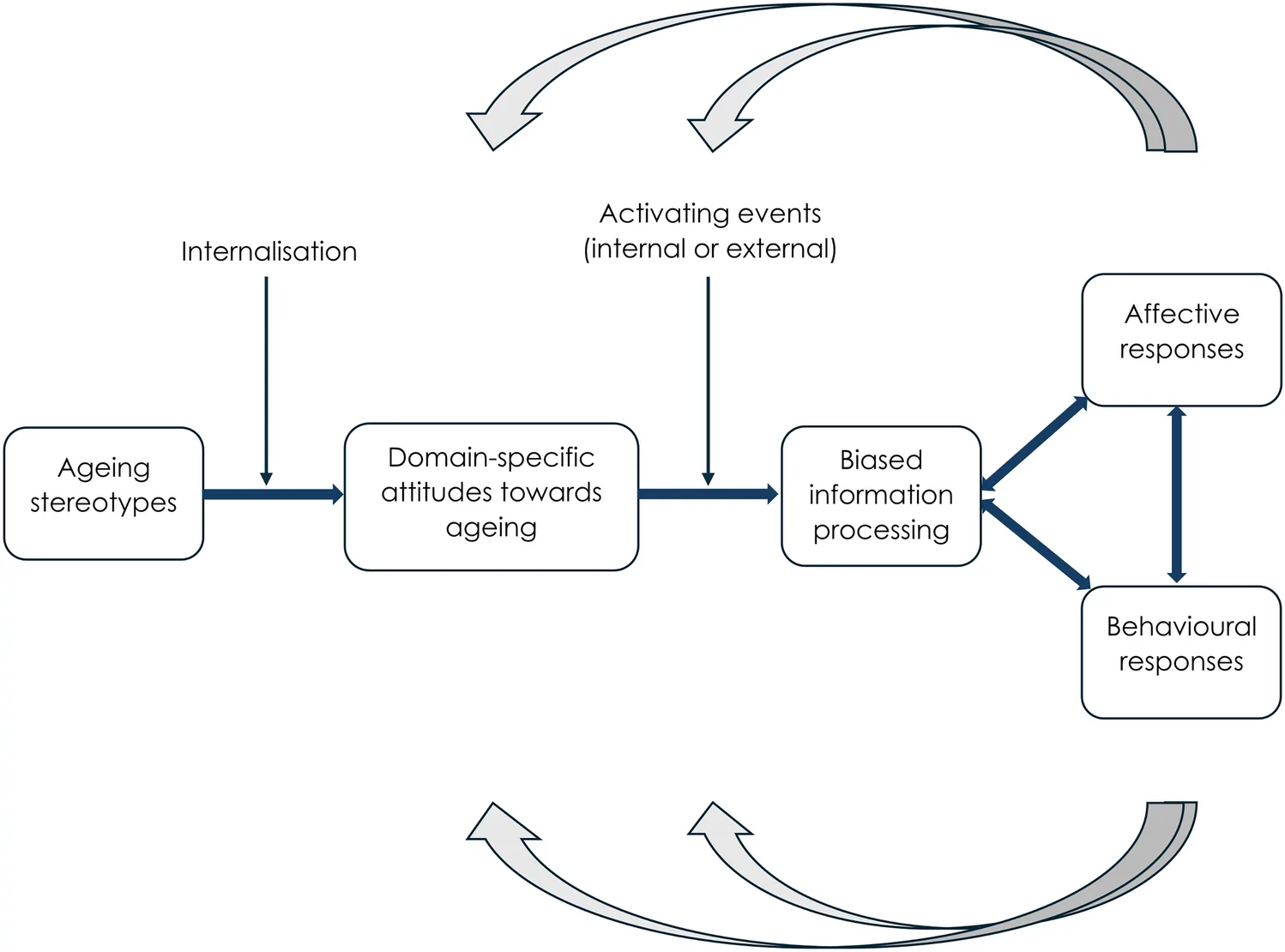
Cognitive model of the stereotype embodiment theory for mental health outcomes

Global demographic trends suggest that the population of older adults is growing rapidly (Leeson 2018), with the number of people aged ≥ 60 years anticipated to be more than double the number of children by 2050 (United Nations 2009). This rise in population ageing has been paralleled by improved life expectancy and health, greater intergenerational contact, and legislation aiming to enhance older adults’ social environments (Levy 2017; Diehl et al. 2021). However, societal changes have not been matched by more favourable attitudes to ageing over time (Wahl et al. 2022), with negative views remaining prevalent across age groups (North and Fiske 2015; Choi et al. 2022). Attitudes to ageing are stable evaluations of ageing that integrate affective, cognitive, and behavioural components (i.e. feelings, beliefs, and behavioural responses; Hess 2006). Crucially, theoretical frameworks suggest that attitudes to ageing play a central role in the development and maintenance of mental health disorders in older adults. The stereotype embodiment theory (SET; Levy 2009) proposes that ageing stereotypes, which are socially shared beliefs about ageing and older people as a group (Wurm et al. 2017), present a risk factor for mental health difficulties in older age. Specifically, SET posits that exposure to age stereotypes in childhood and young adulthood leads to negative views being unquestionably accepted and internalised. Upon reaching old age, these internalised attitudes become self-relevant and may affect health through psychological, behavioural, and physiological responses, namely: (1) self-fulfilling prophecies, in which individuals ‘match’ negative expectations regarding their abilities, (2) reduced engagement with health-promoting behaviours, and (3) heightened physiological stress responses which increase the risk of adverse health outcomes (e.g. cardiovascular events; Levy 2009; Levy and Leifheit-Limson 2009). Laidlaw (2010) suggested that SET can be integrated with cognitive models of psychopathology to explain specific relationships with mental health outcomes (see Fig. 1). The cognitive model views internalised negative attitudes to ageing as a latent vulnerability, which can be triggered by relevant external and internal events (e.g. retirement or physical pain). Once activated, negative attitudes can give rise to cognitive, affective, and behavioural symptoms of mental health disorders. First, information processing is biased towards stimuli which are congruent with the underlying attitudes, leading to changes in attention, memory, and interpretation of events (Disner et al. 2011; Beck and Haigh 2014). For example, expectations of older age as a time of poor health may lead to increased attention to negative physical sensations and attributing these specifically to the ageing process. This distorted information processing results in emotional distress and maladaptive behaviours (Beck and Haigh 2014). For instance, heightened attention to physical changes can increase feelings of anxiety or low mood, whilst interpreting these changes as a normative aspect of ageing may reduce engagement with health-promoting behaviours. These affective and behavioural responses can further worsen physical health and reinforce negative attitudes, resulting in a vicious cycle. Importantly, worldwide estimates suggest that approximately a third of older adults are affected by mental health disorders such as depression and anxiety (Cai et al. 2023; Shafiee et al. 2025). There is thus a clear need for effective interventions to improve older people’s mental well-being. The cognitive approach to the stereotype embodiment theory outlined above indicates that internalised attitudes to ageing may present a risk factor for mental health difficulties. An evaluation of current evidence for this relationship would therefore be valuable for assessing the pathways outlined by the theoretical model and clarifying the potential of attitudes to ageing as an intervention target. Previous reviews (e.g. Westerhof et al. 2023) have typically aggregated results from studies on different elements of views on ageing. Here, views on ageing and (self-)perceptions of ageing are defined as overarching terms which include not only attitudes to ageing, but also related constructs such as awareness of age-related changes (AARC) and subjective age (Wurm et al. 2017; Hausknecht et al. 2020). Although these concepts are interconnected, evidence suggests that they represent distinct, measurable aspects of views on ageing which may differentially affect health outcomes (Brothers et al. 2017; Spuling et al. 2020; Carbone et al. 2025). We hypothesise that these constructs could map onto different components of the cognitive model. For example, AARC may reflect an information processing bias, such that attention to specific experiences (e.g. memory lapses) is heightened and attributed to ageing. Given these theoretical distinctions, this review focussed specifically on attitudes to ageing, which the cognitive model conceptualises as a core antecedent for mental health outcomes.

In addition, there is increasing evidence that attitudes to ageing are multidimensional. That is, attitudes can differ in valence (positive, negative, or neutral) and vary across life domains such as physical health and social networks (Laidlaw et al. 2007; Diehl et al. 2014; Faudzi et al. 2019). However, relationships between individual dimensions of attitudes to ageing and mental health have not been systematically evaluated. A second objective of this review was therefore to explore associations between domain-specific attitudes and older adults’ mental health. Notably, the World Health Organisation (WHO) and positive mental health approaches emphasise that mental health should be defined not only by the absence of psychopathological symptoms, but also the presence of positive indicators of well-being (World Health Organization 2004; Margraf et al. 2024). As well as reviewing the literature on symptoms of depression and anxiety, we therefore evaluated current research on well-being as indicated by older adults’ quality of life (QoL).

## Methods

This review was conducted in accordance with preferred reporting items for systematic reviews and meta-analysis (PRISMA) guidelines (Page et al. 2021; see Supplementary Material). A predefined protocol was registered with PROSPERO (International Prospective Register of Systematic Reviews; CRD42024537940).

### Search strategy

PsycInfo, Embase, Medline, CINAHL, Scopus, and Web of Science databases were searched from inception to February 7, 2026. The search strategy was developed in consultation with a librarian. Titles, abstracts, and topics were searched with Boolean operators using key terms and subject headings including ‘attitudes to ageing’, ‘older adults’, ‘depression’, ‘anxiety’, ‘mental health’, and ‘quality of life’ (see Supplementary Material for the full search strategy). Reference lists from selected papers and existing reviews were screened to identify additional relevant studies.

### Inclusion and exclusion criteria

The study inclusion criteria were (1) peer-reviewed observational studies, (2) published in English, (3) measure of attitudes to ageing included as a predictor variable, (4) measure of depression, anxiety, and/or QoL included as an outcome variable, and (5) results reporting the direct relationship between the predictor and outcome variables within the same individual. If multiple papers reported overlapping or highly related samples, studies were evaluated based on their overall alignment with the inclusion criteria and review aims. Where studies were considered highly comparable, only one paper was retained to avoid duplication and overrepresentation of specific cohorts. In these instances, the paper with the largest sample size was selected for review.

Articles which exclusively focussed on young adults (aged 18–35) or included young adults without reporting results by age group were excluded from the review. Studies which only conducted simple correlations or mediation and moderation analyses without reporting direct associations between attitudes and mental health were not included. Mental health in older adults with particular health conditions or living in residential care can be affected by distinct illness-related psychological processes (Vilhjalmsson 1998; Hudson and Moss-Morris 2019) and environmental or institutional factors (Choi et al. 2008; Chao and Chen 2019). Articles focussing on participants with a specific health condition (e.g. HIV, cancer, or dementia) or not living in the community were therefore excluded. Reviews, case reports, book chapters, commentaries, dissertations, editorials, and conference abstracts were not considered for review.

### Screening and data extraction

Titles, abstracts, and full texts were screened by the first reviewer, with data from selected articles extracted using a pre-defined template. 30% of all results were independently screened and extracted by a second reviewer, with any discrepancies resolved in consultation with a third reviewer. Inter-rater reliability was calculated using Cohen’s kappa statistic, with minimum acceptable agreement set at 0.80 (Altman 1990). Data extracted included author(s), publication year, study design, country, participant demographics, outcome measures, analysis methods, and main statistical results.

### Quality assessment

Methodological quality of included articles was evaluated using the AXIS tool (Downes et al. 2016). Study quality was scored as the total number of items with a ‘yes’ response (max. 20). Studies with a score of > 15 were rated as high quality, 10–15 as moderate quality, and < 10 as low quality (Boos et al. 2019). All studies were appraised by the first reviewer with 30% of selected studies rated independently by a second reviewer. Any differences in ratings were resolved through discussion with the third reviewer.

### Data synthesis

Due to the heterogeneity of instruments used to assess attitudes to ageing, mental health, and QoL, a narrative approach was taken to synthesising the outcomes. Studies were grouped and thematically reported according to the outcome of interest (i.e. depression, anxiety, and QoL) and attitude domains, with all results presented in tables.

## Results

**Fig. 2:**
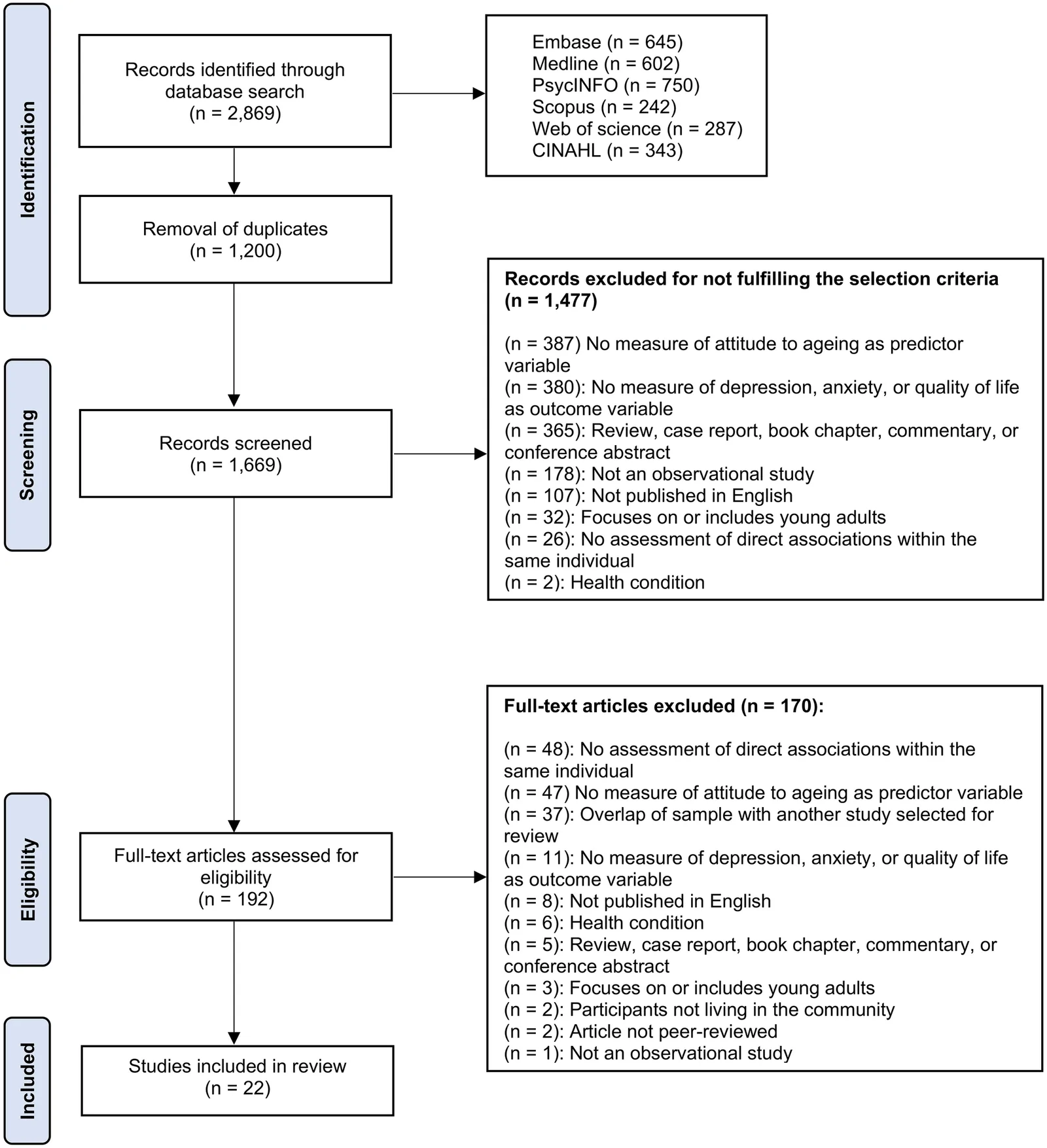
PRISMA flowchart of study selection

The PRISMA flow diagram for study selection is displayed in Fig. 2. The initial search retrieved a total of 2869 publications. Following removal of duplicates, screening of titles and abstracts for 1669 articles was completed. The full texts of 192 papers were reviewed, with 22 studies meeting the inclusion criteria. High inter-rater agreement was reached between the two reviewers completing article selection (κ = 0.83). No additional papers were identified through screening reference lists of included studies and relevant reviews.

### Study characteristics

A summary of the included study characteristics is provided in Table 1 (see Supplementary Material for additional details). The 22 studies reported data from a total of 41,882 individuals, with sample sizes ranging from 40 to 7229 participants. 18 studies employed a cross-sectional design and predominantly used regression-based analytical approaches. Four studies reported longitudinal analyses using panel data from large cohorts, including the Irish Longitudinal Study on Ageing (Freeman et al. 2016), the Health and Retirement Study (Gum and Ayalon 2018), The National Health and Resilience in Veterans Study (Levy et al. 2019), and the German Ageing Survey (Wurm and Benyamini 2014). Longitudinal studies included between two and three measurement waves with follow-up ranging from approximately two to six years. All employed logistic or multiple regression analyses (see Tables 1 and 4–6) and accounted for baseline depression and/or anxiety symptoms. Studies were conducted in Asia (*n* = 9), Europe (*n* = 6), Australia (*n* = 2), and North America (*n* = 3), with two studies reporting data from countries spanning multiple continents (Lai 2009; Long et al. 2021).

**Table 1:** Summary of study characteristics

First author (year)	Design	Country	Max. sample size included in analyses	Age inclusion criterion	Mean age (SD)	Sex (% female)	Outcome(s) of interest
Bryant et al. (2012)	Cross-sectional	Australia	421	≥ 65	71.7 (7.9)	61.5%	Depression Anxiety Quality of life
Chen et al. (2021)	Cross-sectional	China	7229	≥ 60	69.0 (7.4)	45.8%	Depression Quality of lifeL
Choi et al. (2022)	Cross-sectional	South Korea	253	≥ 55	74.0 (6.3)	57.3%	Depression
Chu et al. (2022)	Cross-sectional	Australia	482	≥ 50	61.7 (6.9)	60.2%	Depression Anxiety
Freeman et al. (2016)	Longitudinal (two-year follow-up)	Ireland	6095	≥ 50	63.3 (9.0)	51.7%	Depression Anxiety
Gum and Ayalon (2018)	Longitudinal (four- and six-year follow-up)	USA	4606	≥ 50	65.3 (0.2)	55.5%	Depression
Ingrand et al. (2018)	Cross-sectional	France	258	≥ 55	66.9 (7.9)	46.9%	Quality of life
Kalfoss et al. (2018)	Cross-sectional	Norway	434	≥ 60	75.0 (8.1)	55.8%	Depression
Korkmaz Aslan et al. (2019)	Cross-sectional	Turkey	999	≥ 60	72.3 (23.6)	50.4%	Quality of life
Kornadt et al. (2021)	Cross-sectional	Luxembourg	527	≥ 65	69.9 (7.0)	49.6%	Quality of life
Kulakçı Altıntaş and Ercan Şahin (2025)	Cross-sectional	Turkey	400	≥ 65	Not reported	44.5%	Death anxiety
Lai (2009)	Cross-sectional	China, Hong Kong, Taiwan, Canada, and the USA	4240	≥ 55	69.1 (8.7)	55.5%	Quality of life
Levy et al. (2019)	Longitudinal (two- and four-year follow-up)	USA	2353	≥ 55	68.0 (7.9)	5.5%	Depression Anxiety
Long et al. (2021)	Cross-sectional	20 countries across Europe, North America, South America, Asia, and Africa	4616	≥ 60	72.5 (8.0)	58.5%	Quality of life
Mohammadpour et al. (2018)	Cross-sectional	Iran	330	≥ 60	74.0 (7.7)	57.6%	Death anxiety
Pourghayoumi et al. (2023)	Cross-sectional	Iran	419	60–90	66.9 (6.4)	29.1%	Depression
Sadeghi et al. (2024)	Cross-sectional	Iran	300	≥ 60	68.3 (8.7)	60.7%	Death anxiety
Sindi et al. (2012)	Cross-sectional	Canada	40	58–85	71.3 (1.4)	55.0%	Depression
Sun et al. (2022)	Cross-sectional	China	764	≥ 60	71.8 (7.5)	54.2%	Quality of life
Top et al. (2013)	Cross-sectional	Turkey	550	> 60	68.0 (6.6)	49.1%	Quality of life
Wurm and Benyamini (2014)	Longitudinal (three-year follow-up)	Germany	6205	40–85	61.5 (12.1)	49.5%	Depression
Yamada et al. (2015)	Cross-sectional	Czech Republic	361	≥ 50–91	77.3 (14.0)^a^	66.0%	Quality of life

^a^Interquartile range (IQR)

### Quality assessment

An item-by-item overview of the quality assessment of included studies is provided in Table 2. Eight studies were classified as high quality and 14 as moderate quality. High inter-rater agreement was achieved for the assessment of study quality (κ = 0.80). Few studies justified the sample size (*n* = 5), undertook measures to address and categorise non-responders (*n* = 1), or provided demographic data about non-responders (*n* = 2).

**Table 2:** Quality evaluation of included studies

Author (year)	Aims	Design	Sample size	Population	Sample frame	Selection process	Addressing non-response	Validity measures	Reliability measures	Statistical significance	Methods description	Basic data	Response rate	Description non-response	Internal consistency	Results description	Justified conclusions	Limitations	Funding	Ethical approval	Overall rating
Bryant et al. (2012)	Y	Y	N	Y	Y	Y	N	Y	Y	Y	Y	Y	N	N	Y	Y	Y	Y	Y	Y	High
Chen et al. (2021)	Y	Y	N	Y	U	U	N	Y	Y	Y	Y	Y	U	N	Y	Y	Y	N	U	U	Moderate
Choi et al. (2022)	Y	Y	N	Y	Y	Y	Y	Y	Y	Y	Y	Y	U	N	Y	Y	Y	Y	Y	Y	High
Chu et al. (2022)	Y	Y	N	Y	Y	N	N	Y	Y	Y	N	Y	U	N	Y	Y	Y	Y	Y	Y	Moderate
Freeman et al. (2016)	Y	Y	N	Y	Y	Y	N	Y	Y	Y	Y	Y	Y	N	Y	Y	Y	Y	U	Y	High
Gum and Ayalon (2018)	Y	Y	N	Y	U	Y	N	Y	N	Y	Y	Y	Y	Y	Y	Y	Y	Y	Y	Y	High
Ingrand et al. (2018)	N	Y	N	Y	Y	Y	N	Y	Y	Y	Y	Y	Y	N	Y	Y	Y	N	U	Y	Moderate
Kalfoss et al. (2018)	Y	Y	N	Y	U	U	N	N	Y	Y	Y	Y	N	N	Y	Y	N	N	Y	U	Moderate
Korkmaz Aslan et al. (2019)	Y	Y	Y	Y	Y	Y	N	Y	Y	Y	Y	Y	U	N	Y	Y	Y	Y	U	Y	High
Kornadt et al. (2021)	Y	N	N	Y	N	U	N	Y	N	Y	Y	Y	N	N	Y	Y	Y	Y	Y	Y	Moderate
Kulakçı Altıntaş and Ercan Şahin (2025)	Y	Y	Y	Y	Y	Y	N	Y	Y	Y	Y	N	Y	N	Y	Y	N	Y	Y	Y	High
Lai (2009)	Y	Y	N	Y	U	U	N	N	N	Y	Y	Y	U	Y	Y	Y	N	Y	U	U	Moderate
Levy et al. (2019)	Y	Y	N	Y	Y	Y	N	Y	Y	Y	Y	Y	N	N	Y	Y	Y	Y	N	U	High
Long et al. (2021)	Y	Y	N	Y	U	U	N	Y	Y	Y	N	Y	U	N	Y	Y	Y	N	Y	Y	Moderate
Mohammadpour et al. (2018)	Y	Y	N	Y	Y	N	N	Y	Y	N	Y	Y	U	N	Y	Y	N	N	Y	Y	Moderate
Pourghayoumi et al. (2023)	Y	Y	Y	Y	Y	Y	N	Y	Y	Y	N	Y	Y	N	N	N	N	N	Y	Y	Moderate
Sadeghi et al. (2024)	Y	Y	Y	Y	Y	N	N	Y	Y	Y	Y	Y	U	N	Y	Y	Y	Y	U	Y	Moderate
Sindi et al. (2012)	Y	Y	N	Y	U	U	N	Y	U	Y	Y	N	U	N	Y	Y	Y	Y	Y	Y	Moderate
Sun et al. (2022)	Y	Y	Y	Y	Y	N	N	Y	Y	Y	Y	Y	Y	N	Y	Y	Y	Y	U	Y	High
Top et al. (2013)	Y	Y	N	Y	Y	U	N	Y	Y	Y	Y	Y	N	N	Y	Y	Y	Y	Y	Y	Moderate
Wurm and Benyamini (2014)	Y	Y	N	Y	Y	U	N	Y	U	Y	N	Y	N	N	Y	U	Y	Y	Y	Y	Moderate
Yamada et al. (2015)	Y	Y	N	Y	Y	U	N	Y	Y	Y	Y	N	Y	N	Y	Y	Y	Y	Y	Y	Moderate

*N* = *no; U* = *unclear, Y* = *yes*

### Overview of measures

The most common measures used to assess attitudes to ageing were versions of the Attitudes to Ageing Questionnaire (AAQ; Laidlaw et al. 2007; *n* = 10) and the Ageing Perceptions Questionnaire (APQ; Barker et al. 2007; Sexton et al. 2014; *n* = 5). Other measures used were based on the Philadelphia Geriatric Center Morale Scale (PGCMS; Lawton 1975) Attitudes Towards Own Ageing (ATOA) subscale (*n* = 2), the Ageing Cognition Scales (AgeCog; Steverink et al. 2001; *n* = 2), and the 12-item Expectations Regarding Ageing Survey (ERA-12; Sarkisian et al. 2005; *n* = 2). One article used questions specifically developed for the study (Lai 2009). The PGCMS ATOA subscale and the questionnaire used by Lai (2009) are unidimensional measures, which assess attitudes on a single positive–negative continuum. In contrast, the AAQ, APQ, AgeCog, and ERA-12 contain subscales evaluating different domains of attitudes to ageing. The AAQ and AgeCog distinguish between (psycho)social loss, physical change or decline, and psychological or continuous growth. The ERA-12 assesses ageing-related attitudes towards physical health, mental health, and cognitive function. Finally, the APQ consists of seven subscales which explore individuals’ attitudes towards consequences of ageing (positive versus negative), consistency of awareness of ageing over time (chronic versus cyclical timeline), extent of personal control over ageing processes (positive versus negative), and emotional responses to ageing (Barker et al. 2007). In the brief APQ (B-APQ; Sexton et al. 2014) the negative control and consequences subscales are combined into a single scale and the cyclical timeline subscale is removed. An overview of all attitudes to ageing measures is provided in Table 3.

**Table 3:** Overview of attitudes to ageing measures

Measure	Structure	Nr items	Subscales	Example items
Attitudes to Ageing Questionnaire (AAQ; Laidlaw et al. 2007)	Multidimensional	24	Psychosocial loss Psychological growth Physical change	‘Old age is a time of loneliness’ ‘Wisdom comes with age’ ‘My health is better than I expected for my age’
Ageing Cognition Scales (AgeCog; Steverink et al. 2001)	Multidimensional	12	Social losses Continuous growth Physical decline	‘Ageing means to me… that others don’t need me so much anymore’ ‘Ageing means to me… becoming more and more competent’ ‘Ageing means to me… that I have less physical endurance’
Ageing Perceptions Questionnaire (APQ; Barker et al. 2007)	Multidimensional	32	Consequences positive Consequences negative Control positive Control negative Timeline chronic Timeline cyclical Emotional representations	‘As I get older I get wiser’ ‘As I get older I can take part in fewer activities’ ‘The quality of my social life in later years depends on me’ ‘How mobile I am in later life is not up to me’ ‘I am conscious of getting older all the time’ ‘I go through phases of feeling old’ ‘I feel angry when I think about getting older’
12-item Expectations Regarding Ageing Survey (ERA-12; Sarkisian et al. 2005)	Multidimensional	12	Physical health Mental health Cognitive function	‘Having more aches and pains is an accepted part of ageing’ ‘It’s normal to be depressed when you are old’ ‘I expect that as I get older I will become more forgetful’
Philadelphia Geriatric Center Morale Scale (PGCMS; Lawton 1975) Attitudes Towards Own Ageing subscale	Unidimensional	5	None	‘As you get older, you are less useful’
Lai (2009) attitudes to ageing scale	Unidimensional	6	None	‘Life is all down-slope in old age, and there is not much hope’

Depression was predominantly assessed using versions of the Center for Epidemiological Studies Depression Scale (CES-D; Radloff 1977; *n* = 6) and the Geriatric Depression Scale (GDS; Brink et al. 1982; *n* = 3), with two studies using the 9- or 4-item Patient Health Questionnaire (PHQ-9; Löwe et al. 2004; PHQ-4; Kroenke et al. 2009). General anxiety was measured with the Geriatric Anxiety Scale (GAI; Segal et al. 2010; *n* = 2), the PHQ-4 (Kroenke et al. 2009, *n* = 1), and the Hospital Anxiety and Depression Scale – Anxiety subscale (HADS-A; Zigmond & Snaith 1983; *n* = 1). Two studies explored symptoms of death anxiety using the Death Anxiety Scale (DAS; Templer 1970) and one used the Collett-Lester Fear of Death Scale (CL-FODS; Lester 1990). Most studies used continuous data for analysis of depression and anxiety, with one study applying clinical cut-offs for the CES-D (score ≥ 16) and HADS-A (score ≥ 8) to determine the incidence of depression and anxiety, respectively (Freeman et al. 2016).

Quality of life is a broad concept which the World Health Organisation (The WHOQOL Group 1998, p. 551) defines as “an individual’s perception of their position in life in the context of the culture and value system in which they live and in relation to their goals, expectations and standards and concerns”. Validated instruments capture a range of different domains, including global QoL, health-related QoL, mental QoL, and life satisfaction (Halvorsrud and Kalfoss 2007). Measures used by included studies were the World Health Organisation Quality of Life Module (WHOQOL-OLD; Power et al. 2005; *n* = 4) and abbreviated questionnaire (WHOQOL-BREF; The WHOQOL Group 1998; *n* = 2), the Satisfaction With Life Scale (SWLS; Diener et al. 1985; *n* = 1), the Life Satisfaction Index A (LSI-A; Neugarten et al. 1961; *n* = 1), the CASP-12 (Wiggins et al. 2008; *n* = 1), the Medical Outcomes Study 36-item Short Form (SF-36; Patel et al. 2007; *n* = 1), and the 8-item (SF-8; Ware et al. 2001; *n* = 1) and 12-item Short Form Health Survey (SF-12; Ware et al. 1996; *n* = 1). Two studies used single-item Likert scales to measure QoL and life satisfaction (Chen et al. 2021; Kornadt et al. 2021).

### Attitudes to ageing and depression

Relationships between attitudes to ageing and depression were explored in seven cross-sectional and four longitudinal studies (see Table 4). More negative total attitudes to ageing scores were associated with higher depression scores cross-sectionally (Sindi et al. 2012; Chu et al. 2022; Pourghayoumi et al. 2023) and at 4- and 6-year follow-up (Gum and Ayalon 2018). More negative attitudes to ageing at baseline also predicted incident (Freeman et al. 2016; Levy et al. 2019) and persistent depression (Freeman et al. 2016). Highlighting the importance of the self-relevance of attitudes, Sindi and colleagues (2012) found that negative attitudes towards one’s *own* ageing process but not ageing in general were related to more depressive symptoms.

**Table 4:** Attitudes to ageing and depression

Author (year)	Attitudes to ageing measure	Depression measure	Statistical results	Key findings
Bryant et al. (2012)	Attitudes to Ageing Questionnaire (AAQ)	Centre for Epidemiological Studies Depression Scale (CES-D)	Hierarchical multiple regression: Psychosocial loss and CES-D: *β* = 0.38, *p* < 0.01 Psychological growth and CES-D: depression: *β* = 0.38, *p* < 0.01 Physical change and CES-D: non-significant	Psychosocial loss ↑ depression Psychological growth ↓ depression Physical change Ø depression
Chen et al. (2021)	Attitudes to Ageing Questionnaire (AAQ) – seven-item version with questions from the psychosocial loss and psychological growth domains	Centre for Epidemiological Studies Depression Scale (CES-D) – nine-item version	Hierarchical multiple regression: Psychosocial loss and CES-D: *β* = 0.327, *p* < 0.001 Psychological growth and CES-D: *β* = -0.258, *p* < 0.001	Psychosocial loss ↑ depression Psychological growth ↓ depression
Choi et al. (2022)	Attitudes to Ageing Questionnaire (AAQ)	Center for Epidemiological Studies Depression Scale (CES-D) – ten-item version	Multiple linear regression: Psychosocial loss and CES-D (age 55–64): *β* = − 0.32, *p* < 0.001 Physical change and CES-D (age 55–64): *β* = − 0.04, *p* > 0.05 Psychological growth and CES-D (age 55–64): *β* = − 0.08, *p* > 0.05 Psychosocial loss and CES-D (age ≥ 65): *β* = − 0.27, *p* < 0.001 Physical change and CES-D (age ≥ 65): *β* = − 0.28, *p* < 0.01 Psychological growth and CES-D (age ≥ 65): *β* = − 0.08, *p*-value not clearly reported	*Middle-aged adults:* Psychosocial loss ↑ depression Physical change Ø depression Psychological growth Ø depression *Older adults:* Psychosocial loss ↑ depression Physical change ↑ depression Psychological growth Ø depression
Chu et al. (2022)	12-item Expectations Regarding Ageing Survey (ERA-12)	Geriatric Depression Scale (GDS) with the original dichotomous responses adapted to a four-point likert scale	Independent *t*-test: Total ERA-12 score and GDS: *t*(480) = 7.31, *p* < 0.001	Negative attitudes ↑ depression
Freeman et al. (2016)	Brief Ageing Perceptions Questionnaire (B-APQ)	Center for epidemiological studies depression scale (CES-D)	Multivariate logistic regression: baseline total B-APQ score and CES-D incident depression: OR = 1.09, *p* < 0.001 Baseline total B-APQ score and CES-D persistent depression: OR = 1.05, *p* < 0.01	Negative attitudes ↑ incident and persistent depression
Gum and Ayalon (2018)	Philadelphia Geriatric Center Morale Scale (PGCMS) – Attitude towards own ageing subscale combined with 3 additional questions designed for the study	Center for Epidemiological Studies Depression Scale (CES-D); eight-item version with yes–no response format	Linear regression: Baseline PGCMS subscale and 4-year follow-up CES-D: β = − 0.30, *p* < 0.001 Baseline PGCMS subscale and 6-year follow-up CES-D: β = − 0.41, *p* < 0.001	Negative attitudes ↑ depression
Kalfoss et al. (2018)	Attitudes to ageing questionnaire (AAQ)	Short-form Geriatric Depression Scale (sf-GDS)	Multiple linear regression: Psychosocial loss and sf-GDS: *F*(1419) = 27.74, *p* < 0.001 Physical change and sf-GDS: *F*(1, 421) = 16.52, *p* < 0.001 Psychological growth and sf-GDS: *F*(1419) = 8.97, *p* = 0.003	Psychosocial loss ↑ depression Physical change ↑ depression Psychological growth ↓ depression scores
Levy et al. (2019)	Three-item version of the 12-item Expectations Regarding Ageing Survey (ERA-12)	Patient health questionnaire (PHQ-4)	Logistic regression with category 0 as a referent for comparison with categories 1–3 (representing increasingly negative attitudes): Category 1 and PHQ-4: OR = 2.07, *p* < 0.001 Category 2 and PHQ-4: OR = 2.40, *p* < 0.01 Category 3 and PHQ-4: OR = 2.49, *p* < 0.001	Negative attitudes ↑ incident depression
Pourghayoumi et al. (2023)	Brief ageing perceptions questionnaire (B-APQ)	Patient health questionnaire-9 (PHQ-9)	Hierarchical multiple regression: Total B-APQ score and PHQ-9: *β* = 0.12, *p* < 0.001 Chronic timeline and PHQ-9: OR = 1.12, *p* < 0.001 Emotional representations and PHQ-9: OR = 1.20, *p* < 0.001 Positive consequences and PHQ-9: OR = 0.83, *p* < 0.001 Negative consequences/control and PHQ-9: OR = 0.89, *p* < 0.001	Negative attitudes ↑ depression Chronic timeline ↑ depression Emotional representations ↑ depression Positive consequences ↓depression Positive control ↓ depression Negative consequences and control ↓ depression
Sindi et al. (2012)	8 items assessing general attitudes towards ageing and attitudes towards own ageing, based on the Philadelphia Geriatric Center Morale Scale (PGCMS) – Attitude Towards Own Ageing subscale	Geriatric Depression Scale (GDS)	Stepwise regression: PGCMS subscale self-perceptions and GDS: β = 0.691, *p* = 0.001 PGCMS subscale general perceptions and GDS: non-significant	Negative attitudes to own ageing ↑ depression General negative attitudes Ø depression
Wurm and Benyamini (2014)	Ageing cognition scales (AgeCog) physical decline subscale	Center for Epidemiological Studies Depression scale (CES-D) – 15-item version	Multiple linear regression: AgeCog physical decline and CES-D: *β* = 0.10, *p* < 0.01	Physical decline ↑ depression

↑ Predictor associated with higher outcome scores; ↓ predictor associated with lower outcome scores; Ø no significant association between predictor and outcome variable

For specific domains, higher AAQ psychosocial loss scores were consistently associated with depressive symptomatology (Bryant et al. 2012; Kalfoss et al. 2018; Chen et al. 2021; Choi et al. 2022). Three of these studies found that higher scores on the psychological growth domain were linked with fewer depressive symptoms (Bryant et al. 2012; Kalfoss et al. 2018; Chen et al. 2021), with one study reporting no significant relationship (Choi et al. 2022). Results for attitudes to physical change were mixed. One cross-sectional (Kalfoss et al. 2018) and one longitudinal study (Wurm and Benyamini 2014) indicated that higher physical change and decline scores as measured with the AAQ and AgeCog scales, respectively, predicted more depressive symptoms. However, Bryant et al. (2012) found no significant association and Choi et al. (2022) reported variable outcomes by age group, with higher physical change scores predicting greater depressive symptomatology in older but not middle-aged participants. Only one study reported results from B-APQ domains, showing that stronger negative emotional responses to ageing and higher chronic awareness of ageing scores were linked with more depressive symptoms. In contrast, higher scores on both positive and negative beliefs about the impact of ageing as well as control over positive and negative experiences were associated with lower depression scores (Pourghayoumi et al. 2023).

### Attitudes to ageing and anxiety

Four studies assessed general anxiety and three studies investigated on death anxiety (see Table 5). Two Australian cross-sectional studies suggested that more negative attitudes to ageing on the AAQ and ERA-12 were linked with higher levels of general anxiety (Bryant et al. 2012; Chu et al. 2022). In addition, an Irish longitudinal study demonstrated that total B-APQ baseline scores predicted both incident and persistent anxiety at two-year follow-up (Freeman et al. 2016). Similarly, Levy and colleagues (2019) showed that more negative attitudes on the ERA-12 strongly increased the risk of developing generalised anxiety in a cohort of United States veterans. Specific attitude domains were only assessed by Bryant and colleagues (2012), who reported that higher psychosocial loss scores were linked with more symptoms of anxiety. No significant associations were found for psychological growth and physical change scores. Kulakçı Altıntaş and Ercan Şahin (2025) found that more negative total scores on the Turkish AAQ were associated with greater death anxiety. The other two studies on death anxiety explored associations with domain-specific attitudes on the APQ in Iranian older adults. Mohammadpour et al. (2018) reported that higher chronic awareness and positive consequences of ageing scores were related to less anxiety, whereas higher scores on negative emotional responses to ageing and control over positive experiences predicted more anxiety symptoms. No associations between negative consequences or control over negative experiences and death anxiety were observed. In contrast, Sadeghi et al. (2024) indicated that only higher negative emotional response scores were associated with greater death anxiety.

**Table 5:** Attitudes to ageing and anxiety

Author (year)	Attitudes to ageing measure	Anxiety measure	Statistical results	Key findings
Bryant et al. (2012)	Attitudes to Ageing Questionnaire (AAQ)	Geriatric Anxiety Inventory (GAI)	Hierarchical multiple regression: Psychosocial loss and GAI: *β* = 0.41, *p* < 0.01 Psychological growth and GAI: non-significant Physical change and GAI: non-significant	Psychosocial loss ↑ anxiety Psychological growth Ø anxiety Physical change Ø anxiety
Chu et al. (2022)	12-item Expectations Regarding Ageing Survey (ERA-12)	Geriatric Anxiety Inventory (GAI) with the original dichotomous responses adapted to a four-point Likert scale	Independent *t*-test: Total ERA-12 score and GAI: *t*(480) = 6.47, *p* < 0.001	Negative attitudes ↑ anxiety
Freeman et al. (2016)	Brief Ageing Perceptions Questionnaire (B-APQ)	Hospital Anxiety and Depression Scale – Anxiety subscale (HADS-A)	Multivariate logistic regression: Baseline total B-APQ score and HADS-A incident anxiety: OR = 1.04, *p* < 0.05 Baseline total B-APQ score and HADS-A persistent anxiety: OR = 1.04, *p* < 0.001	Negative attitudes ↑ incident and persistent anxiety
Kulakçı Altıntaş and Ercan Şahin (2025)	Attitudes to Ageing Questionnaire Turkish Version (AAQ-TR)	Death Anxiety scale (DAS)	Linear regression: Total AAQ-TR score and DAS: *β* = − 0.154, *p* = 0.001	Negative attitudes ↑ death anxiety
Levy et al. (2019)	Three-item version of the 12-item Expectations Regarding Ageing Survey (ERA-12)	Patient Health Questionnaire (PHQ-4)	Logistic regression with category 0 as a referent for comparison with categories 1–3 (representing increasingly negative attitudes): Category 1 and PHQ-4: OR = 1.78, *p* < 0.01 Category 2 and PHQ-4: OR = 2.97, *p* < 0.001 Category 3 and PHQ-4: OR = 6.55, *p* < 0.001	Negative attitudes ↑ incident anxiety
Mohammadpour et al. (2018)	Brief Ageing Perceptions Questionnaire (B-APQ)	Collett-Lester Fear of Death Scale (CL-FODS)	Linear regression: Timeline chronic and CL-FODS: *β* = − 0.13, *p* = 0.01 Positive consequences and CL-FODS: *β* = − 0.11, *p* = 0.04 Positive control and CL-FODS: *β* = 0.10, *p* = 0.04 Emotional representations and CL-FODS: *β* = 0.22, *p* < 0.001 Negative consequences/control and CL-FODS: *β* = 0.10, *p* = 0.05	Timeline chronic ↓ anxiety Positive consequences ↓ anxiety Positive control ↑ anxiety Emotional representations ↑ death anxiety Negative consequences and control Ø anxiety
Sadeghi et al. (2024)	Ageing Perceptions Questionnaire (APQ)	Death Anxiety Scale (DAS)	Multiple linear regression: Emotional representations and DAS: *β* = 0.07, *p* = 0.003 All remaining APQ subdomains and DAS: non-significant	Emotional representations ↑ death anxiety Negative control, negative consequences, positive control, positive consequences, chronic timeline, and cyclical timeline Ø DAS

↑ Predictor associated with higher outcome scores; ↓ predictor associated with lower outcome scores; Ø no significant association between predictor and outcome variable

### Attitudes to ageing and quality of life

Ten cross-sectional studies investigated associations between attitudes to ageing and QoL (see Table 6). More negative attitudes to ageing were associated with lower global (Yamada et al. 2015), health-related (Sun et al. 2022), and mental QoL (Lai 2009). Ingrand et al. (2018) combined APQ subdomains into positive and negative dimensions. Higher scores on the positive dimension directly predicted QoL, whereas the negative dimension exerted only indirect effects on QoL mediated by psychological distress.

**Table 6:** Attitudes to ageing and quality of life

Author (year)	Attitudes to ageing measure	QoL measure	Statistical results	Key findings
Bryant et al. (2012)	Attitudes to Ageing Questionnaire (AAQ)	Medical Outcomes 12-item Short Form Health Survey (SF-12); physical and mental subdomains Satisfaction With Life Scale (SWLS)	Hierarchical multiple regression: Psychosocial loss and physical SF-12: *β* = − 0.15, *p* < 0.01 Physical change and physical SF-12: *β* = 0.37, *p* < 0.01 Psychological growth and physical SF-12: *β* = − 0.15, *p* < 0.01). Psychosocial loss and mental SF-12: *β* = − 0.36, *p* < 0.01 Physical change and mental SF-12: *β* =—0.18, *p* < 0.01 Psychological growth and mental SF-12: *β* = 0.10, *p* < 0.05 Psychosocial loss and SWLS: *β* = − 0.13,* p* < 0.01 Psychological growth and SWLS: *β* = 0.26, *p* < 0.01 Physical change and SWLS: non-significant	Psychosocial loss ↓ physical QoL, mental QoL, and satisfaction with life Physical change ↓ physical and mental QoL Physical change Ø satisfaction with life Psychological growth ↓ physical QoL Psychological growth ↑ mental QoL and satisfaction with life
Chen et al. (2021)	Attitudes to Ageing Questionnaire (AAQ) – seven-item version with questions from the psychosocial loss and psychological growth domains	5-point Likert Scale	Hierarchical multiple regression: Psychosocial loss and QoL: *β* = − 0.036, *p* < 0.001 Psychological growth and QoL: *β* = 0.042, *p* < 0.001	Psychosocial loss ↓ QoL Psychological growth ↑ QoL
Ingrand et al. (2018)	Ageing Perceptions Questionnaire (APQ)	CASP-12 Life Satisfaction Index A (LSI-A)	Structural equation model: Positive consequences and control and CASP-12/LSI-A: *β* = 0.39, *p* < 0.001 Negative consequences and control, chronic and cyclical timeline, and emotional representations and CASP-12/LSI-A: non-significant	Positive consequences and control ↑ global QoL
Korkmaz Aslan et al. (2019)	Attitudes to Ageing Questionnaire (AAQ)	WHOQOL-OLD	Hierarchical multiple regression: Psychosocial loss and WHOQOL-OLD: *β* = − 0.289, *p* < 0.001 Physical change and WHOQOL-OLD: *β* = 0.296, *p* < 0.001 Psychological growth and WHOQOL-OLD: *β* = 0.279, *p* < 0.001	Psychosocial loss ↓ older adult QoL Physical change ↓ older adult QoL Psychological growth ↑ older adult QoL
Kornadt et al. (2021)	Ageing Cognition Scales (AgeCog)	4-point likert scale for life satisfaction	Hierarchical moderated regression: Social loss and life satisfaction: *β* = − 0.16, *p* < 0.001 Continuous growth and life satisfaction: *β* = 0.10, *p* = 0.006 Physical decline and life satisfaction: *β* = − 0.06, *p* = 0.140	Social loss ↓ life satisfaction Continuous growth ↑ life satisfaction Physical decline Ø life satisfaction
Lai (2009)	5 statements using a 5-point Likert scale developed for the study	Medical outcomes study 36-item Short Form (SF-36), mental health component	Hierarchical multiple regression: Total attitudes score and mental SF-36: *β* = 0.22, *p* < 0.01	Negative attitudes ↓ mental QoL
Long et al. (2021)	Attitudes to Ageing Questionnaire (AAQ)	WHOQOL-BREF WHOQOL-OLD	Hierarchical multiple regression: Psychosocial loss and WHOQOL-BREF: *β* = 0.242, *p* < 0.001 Physical change and WHOQOL-BREF: β = 0.339, *p* < 0.001) Psychological growth and WHOQOL-BREF: β = 0.032, *p* < 0.01 Psychosocial loss and WHOQOL-OLD: *β* = 0.311, *p* < 0.001 Physical change and WHOQOL-OLD: *β* = 0.207, *p* < 0.001 Psychological growth and WHOQOL-OLD: *β* = 0.121, *p* < 0.001	Psychosocial loss ↓ global and older adult QoL Physical change ↓ global and older adult QoL Psychological growth ↑ global and older adult QoL
Sun et al. (2022)	Attitudes to Ageing Questionnaire (AAQ) – short version	8-item Short Form Health Survey (SF-8)	Multiple linear regression: Total AAQ score and health SF-8:* β* = − 0.211, *p* < 0.01	Negative attitudes ↓ health-related QoL
Top et al. (2013)	Attitudes to Ageing Questionnaire (AAQ)	WHOQOL-OLD	Multiple linear regression: Physical change and WHOQOL-OLD: *β* = 0.185, *p* < 0.001 Psychological growth and WHOQOL-OLD: *β* = 0.378, *p* < 0.001) Psychosocial loss and WHOQOL-OLD: *β* = 0.062, *p* = 0.102	Physical change ↓ older adult QoL Psychological growth ↑ older adult QoL Psychosocial loss Ø older adult QoL
Yamada et al. (2015)	Attitudes to Ageing Questionnaire (AAQ)	WHOQOL-BREF WHOQOL-OLD	Generalised linear model: Total AAQ score and WHOQOL-BREF: *β* = 0.8, *p* < 0.001 Total AAQ score and WHOQOL-OLD: *β* = 0.9, *p* < 0.001	Negative attitudes ↓ global and older adult QoL

↑ Predictor associated with higher outcome scores; ↓ predictor associated with lower outcome scores; Ø no significant association between predictor and outcome variable

Six studies investigated specific attitude domains measured with the AAQ and AgeCog scales. Higher psychosocial loss scores were associated with lower life satisfaction and poorer global, physical, and mental QoL (Bryant et al. 2012; Korkmaz Aslan et al. 2019; Long et al. 2021; Chen et al. 2021; Kornadt et al. 2021), with only one study reporting a non-significant relationship with global QoL (Top et al. 2013). Conversely, higher psychological and continuous growth scores predicted better global QoL and life satisfaction (Korkmaz Aslan et al. 2019; Chen et al. 2021; Kornadt et al. 2021). However, Bryant et al. (2012) suggest psychological growth scores may be differentially related to specific QoL components, with associations being positive for mental QoL and life satisfaction but negative for physical QoL. They hypothesise that a reverse causal relationship may explain the latter finding, such that individuals with more physical health difficulties may develop more effective coping skills which are reflected in psychological growth scores. Results for the physical change domain also varied across QoL measures. Several studies reported that higher AAQ physical change scores predicted lower global, mental, and physical QoL on the WHOQOL-BREF and WHOQOL-OLD (Top et al. 2013; Korkmaz Aslan et al. 2019; Long et al. 2021), which assess a wide range of life domains. However, there was no significant relationship between AAQ or AgeCog physical domain scores and unidimensional measures of life satisfaction (Bryant et al. 2012; Kornadt et al. 2021).

## Discussion

Consistent with the cognitive model integrated with stereotype embodiment theory (Levy 2009; Laidlaw 2010), this systematic review found that attitudes to ageing were associated with older adults’ mental health outcomes. Evidence was strongest for a relationship between negative attitudes and depressive symptoms, which was observed in large-scale cross-sectional and longitudinal studies. Although fewer studies investigated anxiety symptoms, results to date suggest that more negative attitudes predict higher generalised anxiety, with preliminary findings suggesting similar associations with death anxiety. Finally, whilst significant associations with QoL were also consistently reported, the lack of longitudinal research limits inferences regarding the direction of this relationship.

### Components of attitudes to ageing

A second objective of the review was to explore associations between specific attitude domains and older adults’ mental health. The most frequently measured domains using the AAQ and AgeCog scales were social loss, psychological/continuous growth, and physical change. Although the APQ is similarly a multidimensional instrument, the small number of studies reporting results for its individual subscales limits robust conclusions about their relationship with mental well-being.

Social loss scores were consistently associated with all outcomes of interest. This extends research showing that the *experience* of social isolation predicts mental health in older people (Cornwell and Waite 2009; Domènech-Abella et al. 2019) and suggests that *evaluating* older age as a time of social isolation and exclusion could similarly result in poorer mental well-being. The cognitive model predicts that such attitudes may lead to biased processing of socially relevant events and information, such as heightened attention to signs of rejection. This could alter emotional and behavioural responses (e.g. withdrawal or hostility), which in turn may increase social loss and reinforce existing attitudes. In this manner, ageing attitudes could act as a self-fulfilling prophecy in the social domain. In line with this hypothesis, evidence from longitudinal studies indicates that stereotypes and expectations of social changes with age predict subjective feelings of loneliness (Pikhartova et al. 2016) and objective outcomes such as making new friends (Menkin et al. 2017).

In contrast, results showed that viewing older age as a time of growth may serve as a protective factor. It has been proposed that this positive attitude to ageing could increase resilience through developing more adaptive coping strategies (Trică et al. 2024). In accordance with positive psychology and successful ageing perspectives (Ranzijn 2002; Bowling and Iliffe 2011), this highlights the potential of positive attitudes to ageing for increasing older adults’ psychological resources. Of interest, one study in this review suggested that positive and negative attitudes may be differentially linked with mental well-being and psychopathological aspects of mental health, respectively (Ingrand et al. 2018). Additional research is needed to more firmly establish potentially independent effects of positive and negative attitudes to ageing.

Somewhat mixed findings were reported for the relationship between attitudes to physical change and mental health. Whilst some studies indicated that negative attitudes predicted depression and lower QoL, others found that associations were non-significant or varied by age group. Non-significant results were mainly reported by studies using unidimensional measures of attitudes and/or binary outcomes, which were likely less sensitive compared with multidimensional instruments and continuous outcomes. Overall, the findings may reflect a relatively weaker effect of attitudes to physical decline compared with psychological and social change on mental well-being. Levy and Leifheit-Limson (2009) propose a stereotype-matching effect, such that age stereotypes have the greatest impact on corresponding outcome domains. Attitudes to physical change may therefore be more strongly linked with physical (e.g. McLachlan et al. 2020; Diehl et al. 2021) than mental health. However, Boeder and Tse (2021) reported domain-congruent effects for psychological growth but generalised effects of physical decline. There is thus a need to further explore domain-dependent effects across different health outcomes.

### Limitations and future research directions

There were several limitations to the present review and existing evidence base. First, this review evaluated observational research and most identified studies were cross-sectional, which limits conclusions about the causality of reported associations. The relationships are likely bidirectional, with negative attitudes leading to poorer mental health and vice versa. For physical health, it has been shown that the impact of negative attitudes to ageing on health is stronger than the reverse relationship (Wurm et al. 2007; Sargent-Cox et al. 2012). Whether this finding can be generalised to mental health remains to be established and requires additional longitudinal and experimental research.

It should be noted that, whilst the AXIS tool was originally developed for evaluating cross-sectional studies, we used this instrument for all studies included in the review for comparability and ease of interpretation. The quality assessment highlighted that most studies did not justify sample size or addressed non-responses. It is likely that sample sizes were sufficiently large to achieve adequate statistical power, with only one study including fewer than 250 participants. However, response rates varied substantially, with reported rates ranging from 34.9% to 81.3% (see Supplementary Table A) and most studies did not describe non-responders’ demographics. This limitation of the evidence base raises concerns about potential sampling biases, which could affect the generalisability of the findings. In addition, this review aimed to evaluate associations between attitudes and mental health in the general population. People living in care homes or presenting with specific health conditions were therefore excluded, and it is unclear whether the findings can be extrapolated to these groups of older adults. These populations warrant investigation using tailored conceptual frameworks in future studies.

During the screening process, we encountered high variability in definitions of attitudes to ageing, which reflects a wider challenge within the views-on-ageing literature. This review specifically focussed on articles which used instruments that were consistent with the definition of attitudes to ageing specified in the Introduction, and was informed by a previous review of attitudes to ageing measures (Faudzi et al. 2019). However, we acknowledge that these instruments may measure overlapping constructs and vary in the extent to which they assess attitudes related to the self, others, and society (Klusmann et al. 2020). These differences could contribute to variability across findings and highlight the value of greater conceptual and terminological consistency within the literature. The inclusion of additional constructs, such as AARC, subjective age, and essentialist beliefs about ageing, could provide useful information about the contribution of different elements of views on ageing to older adults’ mental health. However, whilst interrelated, these constructs can be conceptually and empirically distinguished from one another and may have different effects on health outcomes (Brothers et al. 2017; Spuling et al. 2020; Carbone et al. 2025). As the theoretical framework outlined in this review emphasises the centrality of attitudes to ageing, we aimed to provide a detailed overview of specific associations between attitudes and older adults’ mental health. However, it would be valuable to examine how attitudes may interact with other views on ageing constructs.

### Clinical implications

This review demonstrates that ageing attitudes are associated with mental well-being in older age, and may present a novel target for both public and individual interventions. From a societal perspective it has been proposed that policies should be developed to challenge negative views of ageing, which could include public education on healthy ageing processes, presenting a balanced view of older age in the media, and providing positive examples of ageing (Lu et al. 2010; Kotter-Grühn 2015; World Health Organization 2015; Tully-Wilson et al. 2021). Such strategies may not only support older adults to adjust their attitudes to ageing, but also serve a preventative function by protecting younger adults from developing negative internalised attitudes before they become self-relevant (Levy 2009; Tully-Wilson et al. 2021).

In addition, specific interventions for addressing negative attitudes to ageing may be developed. Interventions to date have typically adopted group approaches which involve provision of counter-stereotypical information and behaviour change techniques (Knight et al. 2022). Preliminary evidence suggests that such programmes can be effective in reducing negative attitudes to ageing (Burnes et al. 2019; Knight et al. 2022). However, most of these studies were carried out in younger populations (Burnes et al. 2019) or explored effects on physical rather than mental health (Knight et al. 2022). Only one study included depression as an outcome measure, reporting that a group involving exercise combined with an attitudes to ageing intervention reduced average depression scores (Beyer et al. 2019). Further work is needed to determine whether group interventions focussed on changing negative attitudes to ageing can improve mental health and quality of life in older adults.

Finally, the results indicate that adjustments to standard therapeutic protocols may be beneficial for older adults presenting with mental health disorders. Laidlaw (2010) emphasised that, if not addressed by the therapist, negative attitudes to ageing could limit progress in therapy. In addition, as attitudes to ageing may directly affect older people’s mental health, they could form a specific target for therapy. In this context, interventions could aim to both reduce negative attitudes and emphasise growth as a positive aspect of ageing (Laidlaw 2010). As such, it has been suggested that attitudes to ageing questionnaires may be useful as a screening tool for identifying therapy goals and monitoring intervention outcomes (Tully-Wilson et al. 2021). Additional research is needed to determine whether incorporating strategies related to ageing attitudes can enhance standard therapeutic treatments for older adults.

## Conclusion

This review demonstrates that there is a significant relationship between attitudes to ageing and depression, anxiety, and quality of life in older adults. The evidence suggests that negative attitudes to psychosocial loss are linked to poorer mental health, whereas positive attitudes to psychological growth may be a protective factor. Altogether, our findings are consistent with the stereotype embodiment theory and indicate that attitudes to ageing may present a suitable target for interventions aiming to improve older adults’ mental well-being. We propose that a main priority for future research is the use of experimental and interventional approaches to more clearly establish the causality of the observed associations and clinical application of attitudes to ageing as a treatment target.

## Supplementary Information

Below is the link to the electronic supplementary material.

Supplementary file1 (DOCX 32 KB)

## Data Availability

The protocol for this review was preregistered with PROSPERO (https://www.crd.york.ac.uk/PROSPERO/view/CRD42024537940). The data extraction spreadsheet is available on request.
