# Supplementary material for: Associations between older adults’ attitudes to ageing, mental health, and quality of life: a systematic review
Source: Eur J Ageing. 2026 Jul 9;23(1):37. doi: 10.1007/s10433-026-00932-x (PMC13635059; doi:10.1007/s10433-026-00932-x)
Supplement: Supplementary file 1 [file 10433_2026_932_MOESM1_ESM.docx]

**Supplementary Material**

**Article title:** The impact of attitudes to ageing on older adults’ mental health	**Journal name:** European Journal of Ageing	**Author names:** Margot Juliëtte Overman^a^, Lucy Hopkins^a^, and Kathryn Evans^a^	**Affiliation:** ^a^ Oxford Institute of Clinical Psychology Training and Research	**Corresponding author:** Margot Juliëtte Overman, [margot.overman@psy.ox.ac.uk](mailto:margot.overman@psy.ox.ac.uk)	**1. PRISMA checklists**

| **Section and Topic** | **Item #** | **Checklist item** | **Location where item is reported** |
| --- | --- | --- | --- |
| **TITLE** | | |  |
| Title | 1 | Identify the report as a systematic review. | Title |
| **ABSTRACT** | | |  |
| Abstract | 2 | See the PRISMA 2020 for Abstracts checklist. | Abstract |
| **INTRODUCTION** | | |  |
| Rationale | 3 | Describe the rationale for the review in the context of existing knowledge. | p. 3-5 |
| Objectives | 4 | Provide an explicit statement of the objective(s) or question(s) the review addresses. | p. 5 |
| **METHODS** | | |  |
| Eligibility criteria | 5 | Specify the inclusion and exclusion criteria for the review and how studies were grouped for the syntheses. | p. 7-8 |
| Information sources | 6 | Specify all databases, registers, websites, organisations, reference lists and other sources searched or consulted to identify studies. Specify the date when each source was last searched or consulted. | p. 6-7 |
| Search strategy | 7 | Present the full search strategies for all databases, registers and websites, including any filters and limits used. | p. 6-7 and Supplementary Materials |
| Selection process | 8 | Specify the methods used to decide whether a study met the inclusion criteria of the review, including how many reviewers screened each record and each report retrieved, whether they worked independently, and if applicable, details of automation tools used in the process. | p. 7-8 |
| Data collection process | 9 | Specify the methods used to collect data from reports, including how many reviewers collected data from each report, whether they worked independently, any processes for obtaining or confirming data from study investigators, and if applicable, details of automation tools used in the process. | p. 7-8 |
| Data items | 10a | List and define all outcomes for which data were sought. Specify whether all results that were compatible with each outcome domain in each study were sought (e.g. for all measures, time points, analyses), and if not, the methods used to decide which results to collect. | p. 7-8 |
|  | 10b | List and define all other variables for which data were sought (e.g. participant and intervention characteristics, funding sources). Describe any assumptions made about any missing or unclear information. | p. 7-8 and Supplementary Materials |
| Study risk of bias assessment | 11 | Specify the methods used to assess risk of bias in the included studies, including details of the tool(s) used, how many reviewers assessed each study and whether they worked independently, and if applicable, details of automation tools used in the process. | p. 8 |
| Effect measures | 12 | Specify for each outcome the effect measure(s) (e.g. risk ratio, mean difference) used in the synthesis or presentation of results. | N/A |
| Synthesis methods | 13a | Describe the processes used to decide which studies were eligible for each synthesis (e.g. tabulating the study intervention characteristics and comparing against the planned groups for each synthesis (item #5)). | p. 8 |
|  | 13b | Describe any methods required to prepare the data for presentation or synthesis, such as handling of missing summary statistics, or data conversions. | N/A |
|  | 13c | Describe any methods used to tabulate or visually display results of individual studies and syntheses. | p. 8 |
|  | 13d | Describe any methods used to synthesize results and provide a rationale for the choice(s). If meta-analysis was performed, describe the model(s), method(s) to identify the presence and extent of statistical heterogeneity, and software package(s) used. | p. 8 |
|  | 13e | Describe any methods used to explore possible causes of heterogeneity among study results (e.g. subgroup analysis, meta-regression). | N/A |
|  | 13f | Describe any sensitivity analyses conducted to assess robustness of the synthesized results. | N/A |
| Reporting bias assessment | 14 | Describe any methods used to assess risk of bias due to missing results in a synthesis (arising from reporting biases). | p. 8 |
| Certainty assessment | 15 | Describe any methods used to assess certainty (or confidence) in the body of evidence for an outcome. | N/A |
| **RESULTS** | | |  |
| Study selection | 16a | Describe the results of the search and selection process, from the number of records identified in the search to the number of studies included in the review, ideally using a flow diagram. | p. 8 and Figure 2 |
|  | 16b | Cite studies that might appear to meet the inclusion criteria, but which were excluded, and explain why they were excluded. | N/A |
| Study characteristics | 17 | Cite each included study and present its characteristics. | p. 25-27, Table 1, and Supplementary Materials |
| Risk of bias in studies | 18 | Present assessments of risk of bias for each included study. | p. 10 and Table 2 |
| Results of individual studies | 19 | For all outcomes, present, for each study: (a) summary statistics for each group (where appropriate) and (b) an effect estimate and its precision (e.g. confidence/credible interval), ideally using structured tables or plots. | Tables 4-6 |
| Results of syntheses | 20a | For each synthesis, briefly summarise the characteristics and risk of bias among contributing studies. | p. 25-27 and Table 2 |
|  | 20b | Present results of all statistical syntheses conducted. If meta-analysis was done, present for each the summary estimate and its precision (e.g. confidence/credible interval) and measures of statistical heterogeneity. If comparing groups, describe the direction of the effect. | N/A |
|  | 20c | Present results of all investigations of possible causes of heterogeneity among study results. | N/A |
|  | 20d | Present results of all sensitivity analyses conducted to assess the robustness of the synthesized results. | N/A |
| Reporting biases | 21 | Present assessments of risk of bias due to missing results (arising from reporting biases) for each synthesis assessed. | N/A |
| Certainty of evidence | 22 | Present assessments of certainty (or confidence) in the body of evidence for each outcome assessed. | N/A |
| **DISCUSSION** | | |  |
| Discussion | 23a | Provide a general interpretation of the results in the context of other evidence. | p. 27-29 |
|  | 23b | Discuss any limitations of the evidence included in the review. | p. 29-31 |
|  | 23c | Discuss any limitations of the review processes used. | p. 29-31 |
|  | 23d | Discuss implications of the results for practice, policy, and future research. | p. 29-32 |
| **OTHER INFORMATION** | | |  |
| Registration and protocol | 24a | Provide registration information for the review, including register name and registration number, or state that the review was not registered. | p. 6 and p. 34 |
|  | 24b | Indicate where the review protocol can be accessed, or state that a protocol was not prepared. | p. 6 and p. 34 |
|  | 24c | Describe and explain any amendments to information provided at registration or in the protocol. | N/A |
| Support | 25 | Describe sources of financial or non-financial support for the review, and the role of the funders or sponsors in the review. | p. 34 |
| Competing interests | 26 | Declare any competing interests of review authors. | p. 34 |
| Availability of data, code and other materials | 27 | Report which of the following are publicly available and where they can be found: template data collection forms; data extracted from included studies; data used for all analyses; analytic code; any other materials used in the review. | p. 34 |

**2. Search strategy**

The following keyword search was applied across databases:

*attitude* to* ag?ing OR attitude* to* aged OR attitude* to* own ag?ing OR ag?ing attitude* OR perception* of ag?ing OR ag?ing perception* OR ag?ing self?perception* OR self*perception* of ag?ing OR perception* of older adult* OR belief* about ag?ing OR ag?ing belief**

*AND*

*depress* OR low mood OR mood disorder* OR affective disorder* OR anxiety OR anxious OR mental health OR mental well?being OR psychological well?being OR subjective well?being or mental well?being OR quality of life OR life satisfaction*

*AND*

*old* adult* OR old* age OR old* people OR elderly OR ag?ing adult* OR late adulthood OR late life OR aged*

Where possible, search results were then combined with the following MeSH term searches:

| **Attitudes to ageing** | **Mental health and quality of life** | **Older adults** |
| --- | --- | --- |
| Attitude to ageing  Aged (attitudes toward)  Aging (attitudes toward)  Attitude  Aging | Depression  Major depression  Affective disorders  Anxiety disorders  Anxiety  Mixed anxiety and depression  Mood disorder  Mental health  Quality of life  Life satisfaction  Subjective wellbeing  Psychological wellbeing  Emotional wellbeing | Older adulthood  Aged |

**3. Additional study characteristics**

**Table S1.** Summary of additional study characteristics

| **First author (year)** | **Response rate** | **Inclusion criteria** | **Education** |
| --- | --- | --- | --- |
| Bryant (2012) | 34.9% | Age ≥60 years | Not reported |
| Chen (2021) | 62.8% | Age ≥60 years Score ≥3 on 5 questions from MMSE | M = 6.87 years SD = 4.43 years |
| Choi (2022) | Not reported | Age ≥55 years | Scale of 1-5: M = 3.28 SD = 1.09 |
| Chu (2022) | Not reported | Age ≥50 years | Without post-secondary education: 55.8% |
| Freeman (2016) | 62.0% | Age ≥50 years Not living in an institution No dementia diagnosis | Primary: 1,604 participants Secondary: 2,517 participants Tertiary: 1,966 participants |
| Gum (2017) | Not reported | Age ≥50 years | M = 13.1 years SE = 0.09 years |
| Ingrand (2018) | 70% | Age ≥55 years Not living in nursing home No evidence of cognitive impairment (MMSE score ≥24) | Less than baccalaureat: 66.5% |
| Kalfoss (2018) | 53.1% | Age ≥60 years Able to speak Norwegian No illness likely to cause death within 6 months  No significant cognitive impairment | Less than post-secondary: 62.0% |
| Korkmaz Aslan (2019) | Not reported | Age ≥65 years Literate in Turkish  No communication or cognitive impairment | Illiterate: 12.9% Literate: 28.0% Primary school: 30.0% Secondary school: 15.6% High school: 10.1% University: 3.3% |
| Kornadt (2021) | 27.% via random digit dialling 40% via online surveys | Age ≥60 years | Tertiary or more: 29.5% |

**Table S1 (continued)** Summary of additional study characteristics

| **First author (year)** | **Response rate** | **Inclusion criteria** | **Education** |
| --- | --- | --- | --- |
| Kulakçı Altıntaş (2025) | 90.9% | Age ≥65 years  Living in the community  No verbal communication disabilities Not dependent on others for daily living activities | Illiterate: 17.7%  Literate: 21.5%  Elementary school: 28.0%  Secondary school: 10.5%  High school: 17.7%  College/faculty: 4.3%  Postgraduate: 0.3% |
| Lai (2009) | Mainland China: 68.7% Hong Kong: 21.7% Taiwan: 63.6%  Canada: 77%  USA: not reported | Age ≥55 years Chinese ethnicity | No formal education: 14.5% Primary: 28.2% Secondary: 36.8% Post-secondary and above: 20.5% |
| Levy (2019) | 94% | Age ≥55 years Served on active duty in the United Staes Armed Forces, Military Reserves, or National Guard  Negative screen for PTSD, suicidal ideation, generalised anxiety, and major depressive disorder at baseline | Attended college: 84.4% |
| Long (2021) | Not reported | Age ≥60 years | Illiterate: 141 School/trade: 3,627 College or higher: 1,536 Other: 115 |
| Mohammadpour (2018) | Not reported | Age ≥60 years No recorded history of drug abuse No consumption of psychiatric drugs No existing diagnosis of terminal illness No cognitive impairment that would result in failure to understand questions and answers | Illiterate: 54.2%  Reading and writing: 25.5% Elementary school certificate: 21.1% Middle school certificate: 3.9% High school diploma: 3.3% College/seminary degree: 0.9% |
| Pourghayoumi (2023) | Not reported | Age 60-90 years Permanent residence in the city of Bushehr Able to communicate verbally Living in the community No cognitive impairment | Illiterate: 33.7% Primary school: 26.3% Secondary school: 31.7% Academic education: 8.4% |

**Table S1 (continued)** Summary of additional study characteristics

| **First author (year)** | **Response rate** | **Inclusion criteria** | **Education** |
| --- | --- | --- | --- |
| Sadeghi (2024) | Not reported | Age ≥60 Not using psychiatric medication Not suffering from a terminal disease No cognitive disorder preventing understanding of questions | Illiterate: 43.3%  Primary school: 31.3% Secondary/middle school: 11.3%  High school diploma or pre-university: 10.0% Academic: 4.0% |
| Sindi (2012) | Not reported | No history of head injury, trauma, or cerebral vascular incident No alcohol abuse No medications that have a potential impact on cortisol secretion or cognitive performance No self-report of any general anesthesia in the previous year No signs of dementia or MMSE score <24 | M = 14.8 years  SD = 0.6 years |
| Sun (2022) | 81.3% | Age ≥60 City resident for more than 6 months Able to communicate in Chinese No diagnosis of dementia No serious hearing impairments | Less than primary: 23.0%  Primary: 35.7%  Middle school: 20.6%  High school: 13.0%  Higher education: 7.3% |
| Top (2013) | 49.1% | Age ≥65 years Living at home without community health care No illness that was likely to cause death within the next year No significant cognitive impairment | Illiterate: 242 participants  Primary school: 217 participants  Secondary school: 50 participants  College: 28 participants  Pre-bachelor: 7 participants  Bachelor: 6 participants |
| Wurm (2014) | Baseline: 35.7% Follow-up: 64.4% | Not reported | Divided into categories of low (1), medium (2), and high (3):  M = 2.2  SD = 0.6 |
| Yamada (2015) | Not reported | Age ≥50 years Client of a home care agency  No cognitive impairment | Primary: 31% Vocational: 30% Secondary: 30% University: 9% |
